# Supplementary material for: Splice-Junction-Based Mapping of Alternative Isoforms in the Human Proteome
Source: Cell Rep. Author manuscript; Available in PMC 2020 Jan 15. (PMC6961840; doi:10.1016/j.celrep.2019.11.026)
Supplement: 2 [file NIHMS1546469-supplement-2.zip › DF1/PXD010154/Liver/Liver_8_CLTCL1_LQEHFQLQNLGINPANIGFSTLTMESDK.pdf]

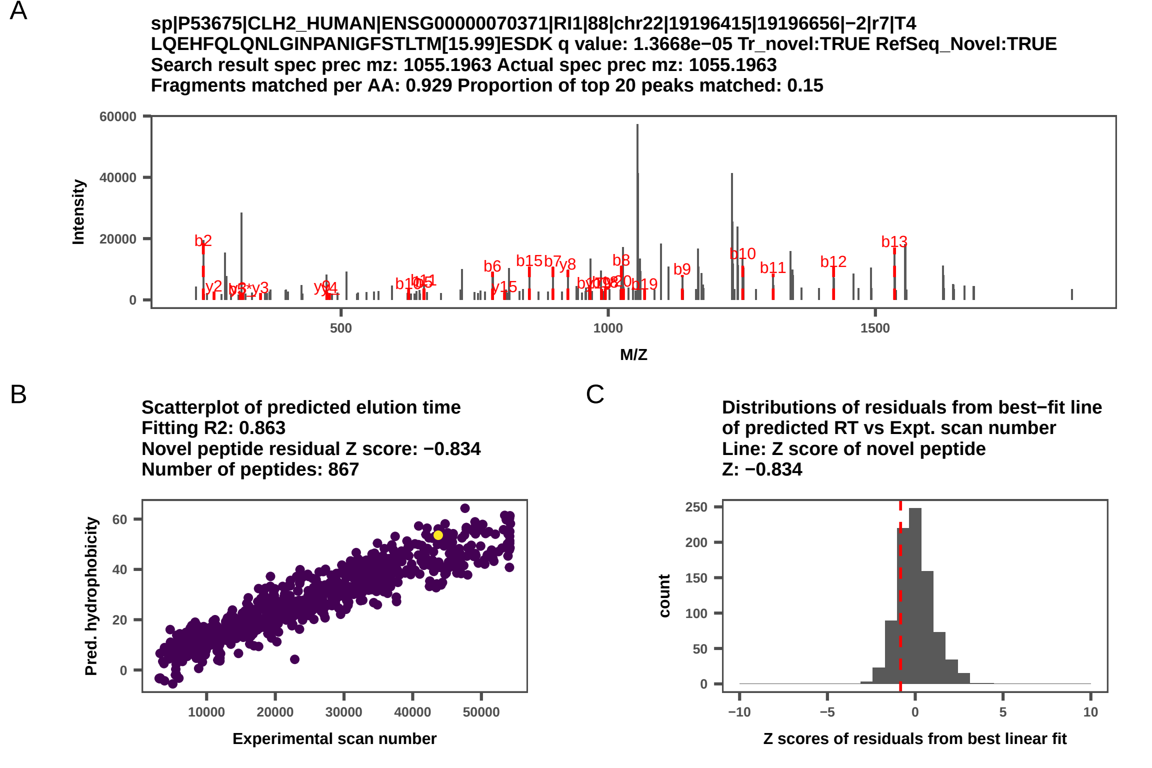
A
sp|P53675|CLH2_HUMAN|ENSG00000070371|RI1|88|chr22|19196415|19196656|−2|r7|T4
LQEHFQLQNLGINPANIGFSTLTM[15.99]ESDK q value: 1.3668e−05 Tr_novel:TRUE RefSeq_Novel:TRUE
Search result spec prec mz: 1055.1963 Actual spec prec mz: 1055.1963
Fragments matched per AA: 0.929 Proportion of top 20 peaks matched: 0.15
Intensity
60000
40000
20000
0
b2
y2
b6
y15
b15
b7
y8
b8
b9
b10
b11
b12
b13
b19
500
1000
1500
M/Z
B
Scatterplot of predicted elution time
Fitting R2: 0.863
Novel peptide residual Z score: −0.834
Number of peptides: 867
Pred. hydrophobicity
60
40
20
0
10000
20000
30000
40000
50000
Experimental scan number
C
Distributions of residuals from best−fit line
of predicted RT vs Expt. scan number
Line: Z score of novel peptide
Z: −0.834
count
250
200
150
100
50
0
−10
−5
0
5
10
Z scores of residuals from best linear fit
